# Circadian Rhythms in Colorectal Cancer: Recent Advances in Development and Treatment

**DOI:** 10.14740/jocmr6626

**Published:** 2026-07-31

**Authors:** Yi Liu, Ting Wang, Rong Jun Zhu, Yu Li Zhang, Hua Sui

**Affiliations:** a Jiading Branch of Shanghai General Hospital, Shanghai Jiao Tong University School of Medicine, Shanghai 201803, China; b The Second Clinical Medical College of Henan University of Chinese Medicine, Zhengzhou, 450000, China; c Nanxiang Branch of Ruijin Hospital, Shanghai Jiao Tong University School of Medicine, Shanghai, 201802, China; d These authors contributed equally to this work.

**Keywords:** Circadian rhythm, Biological clock, Colorectal cancer, Immune microenvironment, Metabolism, Traditional Chinese medicine

## Abstract

The circadian clock orchestrates biological rhythms across species and is deeply involved in the physiological and biochemical processes underlying tumor initiation and progression. By regulating cell division, DNA repair, immune function, and hormonal balance, circadian rhythms interact closely with cancer, providing a theoretical foundation for the exploration of chronotherapy. Maintaining circadian homeostasis is therefore essential for cancer prevention and treatment. This review centers on the intricate relationship between circadian rhythms and malignancy, with a particular focus on the role of circadian disruption in tumorigenesis and malignant progression. A systematic literature search was performed across the PubMed, MEDLINE, and Scopus databases to screen original studies and review articles focusing on circadian regulation in colorectal cancer. This review delineates the tissue-specific effects of circadian clock signaling in colorectal malignancy and clarifies the correlation between circadian disruption and early tumorigenesis. Accumulated evidence is integrated into a comprehensive mechanistic framework covering metabolic reprogramming, tumor immunosuppression, and stromal remodeling. Additionally, current advances in harnessing circadian rhythms to refine preventive strategies and chronotherapeutic regimens for colorectal cancer are highlighted, with a concise discussion on the potential of traditional Chinese medicine in regulating biological rhythms to improve colorectal cancer clinical management.

## Introduction

Circadian rhythms are endogenous biological timing systems with an approximately 24-h cycle [1]. Through a hierarchical network consisting of the central clock in the suprachiasmatic nucleus (SCN) of the hypothalamus and peripheral clocks, they coordinate physiological processes such as the sleep–wake cycle, hormone secretion, metabolism, and immune function [2]. Circadian rhythm disruption (CRD) occurs when the endogenous biological clock becomes misaligned with external environmental cycles or when intrinsic regulatory mechanisms of the clock are impaired [3]. In the context of tumor biology, CRD manifests not only as behavioral disturbances in daily activity patterns but also as dysfunction of core clock genes at the molecular level, including *BMAL1*, *CLOCK*, *PER*, and *CRY* [4]. Such alterations interfere with cellular processes such as cell-cycle regulation, DNA repair, metabolic reprogramming, and immune surveillance, thereby promoting tumor initiation and progression [5].

The etiological factors of CRD can be broadly categorized as exogenous or endogenous. Exogenous contributors primarily include shift work, nighttime light exposure, and irregular eating patterns [6]. Long-term shift work has been associated with an approximately 1.64-fold increase in colorectal cancer (CRC) risk, while exposure to light at night has been associated with increased cancer susceptibility by suppressing melatonin secretion and upregulating CLOCK/brain and muscle ARNT like 1 (BMAL1) expression [7]. Endogenous factors involve genetic variations in core clock genes, abnormal epigenetic modifications, or dysregulated gene expression, all of which weaken the oscillatory function of the molecular clock [8]. Moreover, evidence from the literature indicates that endogenous and exogenous influences interact synergistically, as environmentally induced circadian disruption can further exacerbate functional inactivation of clock genes.

To identify relevant literature for this review, we systematically searched PubMed, MEDLINE, and Scopus databases covering the period from 2020 to 2026. The search strategy incorporated keywords related to circadian rhythms (“circadian rhythm,” “biological clock,” “clock genes,” “chronotherapy”) and colorectal cancer (“colorectal cancer,” “CRC,” “colon cancer,” “rectal cancer”), along with additional terms for specific themes including “tumor microenvironment,” “immunity,” “metabolism,” and “traditional Chinese medicine.” We limited our search to peer-reviewed original research articles, reviews, and clinical studies published in English. Conference abstracts and case reports were excluded. We screened titles and abstracts, and full texts of potentially eligible articles were assessed against the inclusion criteria. Additional relevant studies were identified by manual screening of reference lists of included reviews and original articles. The final selection was based on relevance to the scope of this review, with emphasis on studies addressing circadian regulation of the tumor microenvironment (TME), clinical associations, and therapeutic interventions in CRC.

## Clinical Associations Between Circadian Rhythm and CRC

CRD is closely associated with both the risk of CRC development and patient prognosis [9]. This association is corroborated by evidence from epidemiological cohort studies, as well as genetic analyses.

With regard to disease risk, shift work remains the most extensively studied exogenous factor that perturbs circadian rhythms. The International Agency for Research on Cancer has classified night-shift work as a group 2A probable carcinogen [10]. A prospective cohort study involving African American women demonstrated that long-term night-shift work (≥ 10 years) was significantly associated with an elevated risk of CRC [11]. Systematic reviews and meta-analyses have consistently indicated that shift workers face a higher risk of developing CRC than daytime workers [12]. From a genetic standpoint, Mendelian randomization studies have suggested that individuals genetically predisposed to a “morning chronotype” have a reduced risk of CRC, implying that endogenous circadian preferences may influence susceptibility to CRC [13]. Associations between abnormal sleep duration and CRC risk have been examined in multiple cohort studies, although the findings remain inconsistent [14]. Taken together, these studies suggest that circadian homeostasis confers a protective effect against CRC development.

With respect to survival outcomes, aberrant expression of core clock genes is closely linked to the prognosis of CRC patients. Period (PER)3 expression was found to be significantly downregulated in CRC tissue samples, and low expression correlated with high-grade tumors in clinical cohorts [15]. Beyond PER3, PER1 and PER2 play equally if not more critical roles in circadian regulation and CRC predisposition. Expression profiling studies have demonstrated that PER1, PER2, and PER3 are all markedly downregulated in CRC tumor tissues compared with matched healthy mucosa [16]. PER1 downregulation has been correlated with poor survival and increased incidence of liver metastases. In Apc^Min/+^ mouse models, Per1 deficiency considerably increases intestinal polyp formation and elevates β-catenin protein levels through non-transcriptional mechanisms, supporting a tumor-suppressive role of Per1 in colorectal tumorigenesis [17]. *PER2* mutation increases intestinal β-catenin levels and colon polyp formation, and Per2/Per1 cooperatively regulate β-catenin and cell proliferation in colon cancer cells [18]. Moreover, *Per1* and *Per2* are the major genes regulating the 24-h timekeeping machinery, modulating proliferative, apoptotic, inflammatory, and metastatic signaling. Polymorphism studies have also investigated the association of *PER2* variants with CRC susceptibility, although findings have been inconsistent across populations [19]. Collectively, these findings underscore that PER1 and PER2 are not merely redundant members of the Period family but are central players in both circadian rhythm maintenance and CRC pathogenesis. In contrast, elevated NR1D1 expression has been associated with poor prognosis in CRC patient cohorts [20]. Prognostic models constructed based on circadian clock gene expression profiles have revealed that high expressions of genes such as *CSNK1D*, *NR1D1*, and *CRY2* correlate with shorter disease-free survival in CRC cohorts [21]. Additionally, melatonin levels were found to be markedly reduced in CRC patients and were independently associated with prognosis in clinical cohort studies; melatonin supplementation has been reported to reduce CRC incidence among night-shift workers [22, 23].

In summary, CRD influences both the development and prognosis of CRC through two major pathways: exogenous circadian disturbances and dysregulation of endogenous circadian genes. It suggests that monitoring and regulating circadian rhythms may have potential value in the prevention and prognostic assessment of CRC.

## Regulation of the TME in CRC by Circadian Rhythms

The core molecular mechanism of circadian rhythms, the BMAL1/CLOCK-driven transcription-translation feedback loop, is widely dysregulated in CRC [24]. This dysregulation does not occur solely within tumor cells; rather, it systematically promotes tumor progression and metastasis by reshaping multiple dimensions of the TME [25, 26].

### Metabolic microenvironment

**Figure 1:**
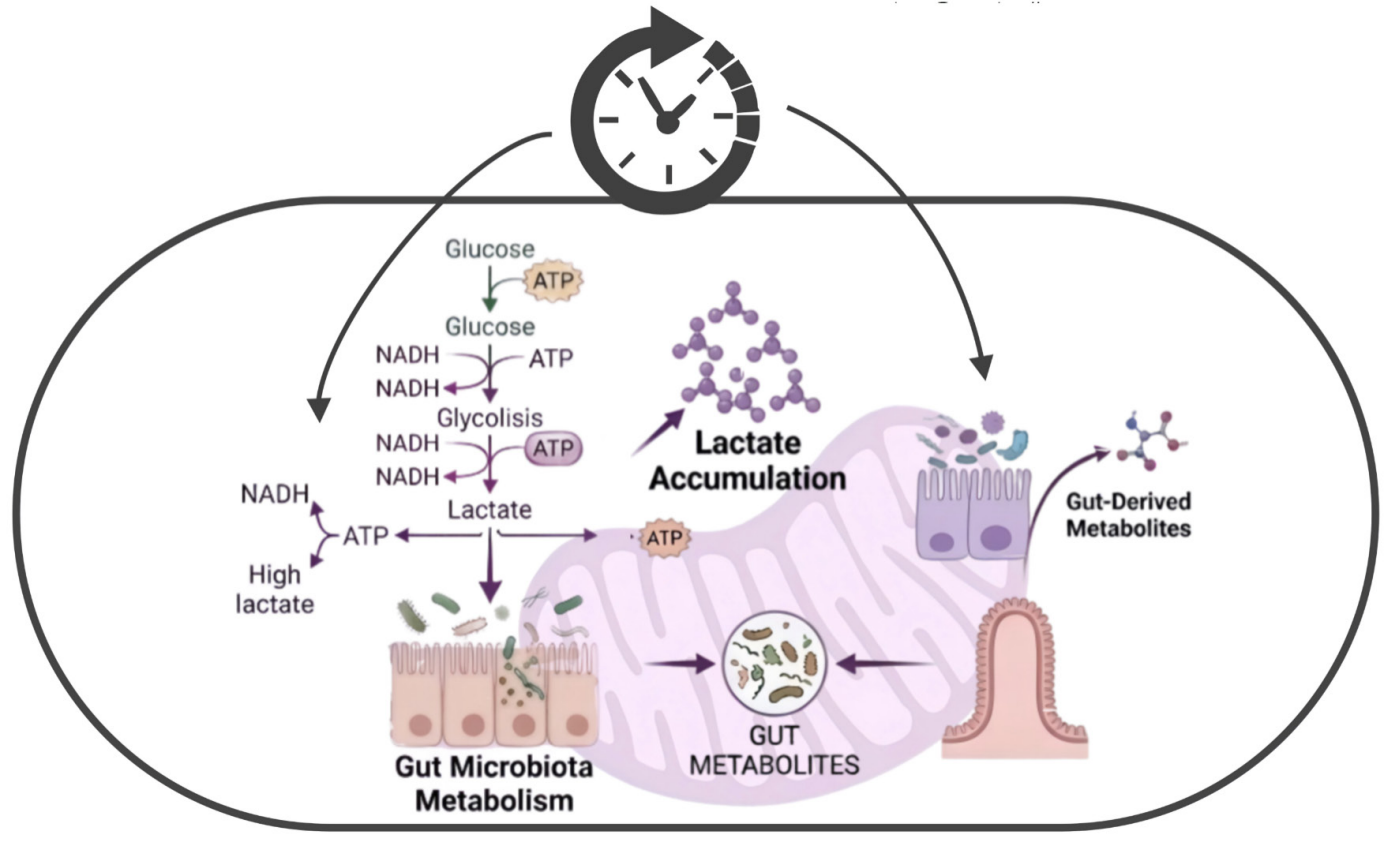
Circadian disruption drives metabolic reprogramming in the CRC TME. Overview of the glycolysis/lactate accumulation pathway driven by BMAL1/PER2 loss, highlighting the suppression of CD8^+^ T cells and promotion of MDSCs. CRC: colorectal cancer; TME: tumor microenvironment; MDSCs: myeloid-derived suppressor cells. BMAL1: brain and muscle ARNT like 1; CRC: colorectal cancer; NADH: reduced nicotinamide adenine dinucleotide; ATP: adenosine triphosphate.

Disruption of circadian rhythms directly drives metabolic reprogramming in the CRC TME through aberrant expression of clock genes [24, 27]. For instance, Chun et al demonstrated that under circadian disruption, Apc loss of heterozygosity hyperactivates Wnt signaling, which in turn upregulates c-Myc and enhances glycolysis, providing direct evidence that circadian misregulation governs glucose metabolism [28]. Further studies have revealed that deficiency of core clock genes, particularly *BMAL1*, *PER2*, and *CRY2*, activates the MYC/hypoxia inducible factor 1 alpha (HIF-1α) signaling axis, thereby driving glycolytic reprogramming and promoting massive lactate accumulation within the TME [28]. Beyond its role as a glycolytic end-product, lactate functions as a signaling molecule that directly acts on immune cells [29]. It suppresses the antitumor activity of CD8^+^ T cells while promoting the immunosuppressive phenotypes of myeloid-derived suppressor cells (MDSCs) and tumor-associated macrophages (TAMs) [30], indicating that circadian disruption extends beyond tumor cell-intrinsic metabolic alterations to reshape the immune microenvironment. Concurrently, circadian disruption alters gut microbiota composition, leading to aberrant accumulation of microbial metabolites such as taurocholic acid (TCA), which in turn epigenetically enhances MDSC glycolysis and inhibits ubiquitin-mediated programmed death ligand 1 (PD-L1) degradation, further unraveling the mechanisms by which circadian disruption drives immune suppression [31]. Collectively, these lines of evidence delineate a vicious cycle in which circadian disruption leads to metabolic reprogramming, which in turn promotes immunosuppression [4], highlighting that the circadian clock serves as a central hub integrating the various components of the TME (Fig. 1).

### Immune microenvironment

Circadian regulation of the immune microenvironment is first reflected in the rhythmic accumulation of immunosuppressive cells. Studies in mouse models of CRC demonstrate that PD-L1^+^ MDSCs reach their maximum abundance in a circadian fashion, with this oscillation being determined by the intrinsic clock of epithelial cells [32].

The function of CD8^+^ T cells also exhibits circadian oscillations. In both murine and human cancers, tumor-infiltrating CD8^+^ T cells display time-of-day-dependent fluctuations in number and function, driven by leukocyte-intrinsic clocks and endothelial cell-dependent rhythmic infiltration. The distribution of CD8^+^ T cells within the TME further varies with time of day, accompanied by diurnal expression of CXCR4 [33].

Dendritic cells (DCs) serve as a critical bridge between innate and adaptive immunity, and their migratory activity and T-cell priming capacity are both subject to circadian regulation. The intrinsic circadian clocks of DCs and CD8^+^ T cells act in concert to govern the time-of-day-dependent patterns of tumor immune infiltration and the efficacy of immunotherapeutic interventions [34]. Mechanistically, the endogenous clock within DCs orchestrates Cx3cl1 expression, thereby facilitating the recruitment of CX3CR1^+^ CD8^+^ T cells into the TME and subsequently remodeling its immunological landscape.

At the molecular level, the circadian clock also contributes to the regulation of immune checkpoint expression. CLOCK promotes PD-L1 expression by mediating acetylation of the NF-κB p65 subunit, whereas DEC2 rhythmically suppresses Pdcd1 transcription, thereby governing PD-1 expression on TAMs. PER2, in contrast, alleviates immune suppression through inhibition of the IKK/NF-κB signaling axis and concomitant downregulation of PD-L1 [35, 36].

*In vivo* studies have shown that the infiltration of immune cells into tumors and their cytotoxic activity fluctuate rhythmically over time, with endothelial cells playing a critical regulatory role in this circadian phenomenon. Macrophages are likewise subject to phase-dependent circadian regulation in experimental systems; their phagocytic activity, cytokine secretion, and M1/M2 polarization states display daily oscillations [37–39]. Furthermore, recent studies have systematically elucidated the emerging mechanisms by which circadian rhythms regulate the gut microbiota and its metabolites—including short-chain fatty acids (SCFAs) and bile acids (BAs)—thereby modulating the function of immune cells such as CD8^+^ T cells, regulatory T cells (Tregs), TAMs, and MDSCs, as well as vascular remodeling within the TME [37]. In particular, it indicates that circadian rhythms affect not only the abundance of immune cells but also their functional states. Taken together, these studies suggest that circadian disruption can lead to excessive accumulation of immunosuppressive cells and functional exhaustion of effector T cells, thereby establishing a temporally defined “immunosuppressive niche” within the TME [37].

**Figure 2:**
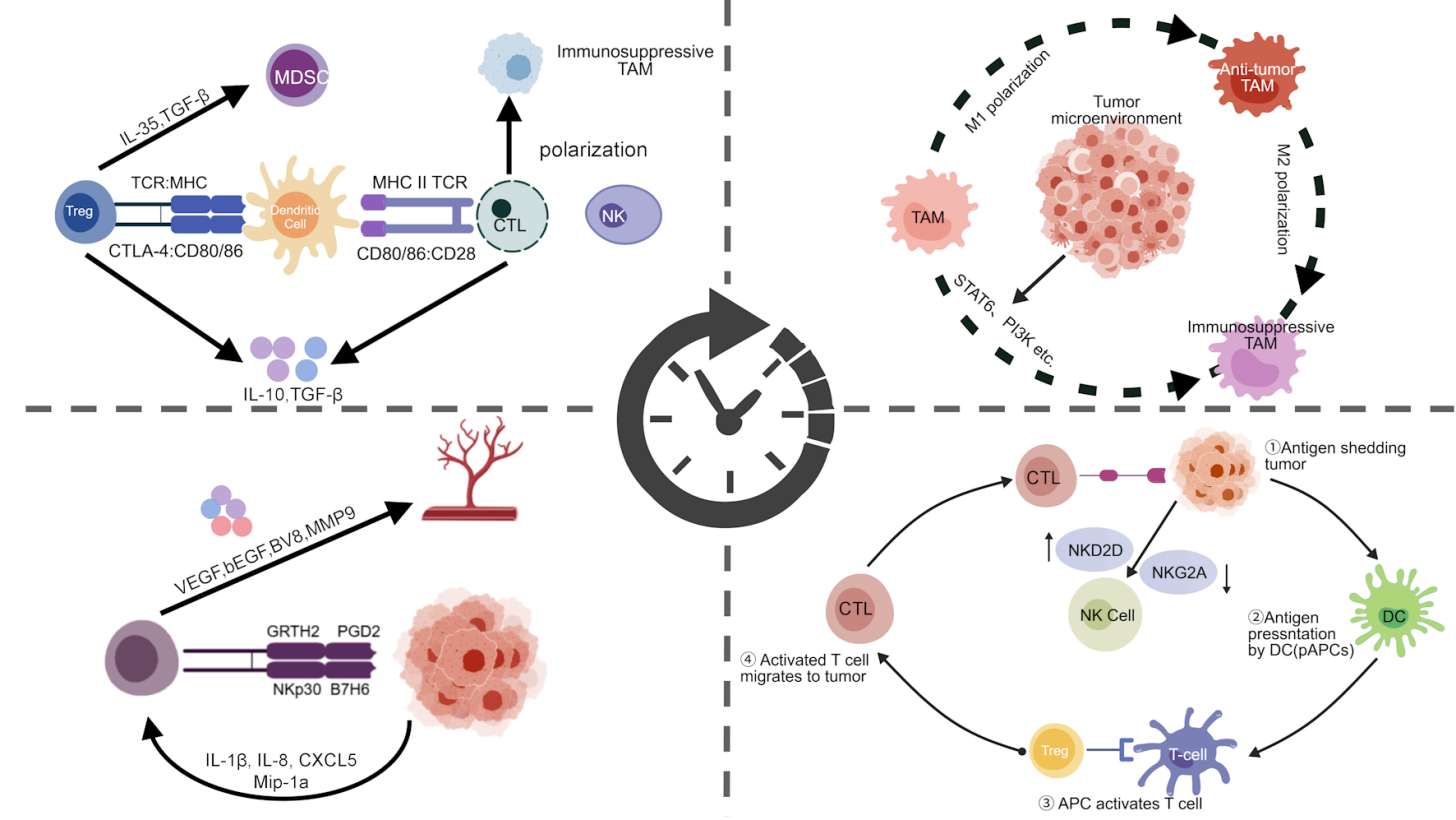
Circadian regulation of the immune microenvironment and its impact on CRC. Schematic of circadian-regulated PD-L1^+^ MDSC abundance and CD8^+^ T cell infiltration, showing the temporal window for anti-PD-L1 therapy. PD-L1: programmed death ligand 1; anti-PD-L1: anti programmed death ligand 1 antibody; CRC: colorectal cancer; TAM: tumor-associated macrophage; MDSC: myeloid-derived suppressor cell; IL: interleukin; VEGF: vascular endothelial growth factor; Treg: regulatory T cell; CTL: cytotoxic T lymphocyte; NK: natural killer cell.

Circadian dysregulation of the immune microenvironment further influences the stromal-physical microenvironment through cytokines secreted by immune cells. Preclinical studies have shown that activated immune cells release factors such as transforming growth factor-beta (TGF-β) and interleukin (IL)-6, which can directly activate cancer-associated fibroblasts (CAFs), promoting their transition toward a myofibroblastic phenotype, and simultaneously stimulate endothelial cell proliferation and angiogenesis [40]. This mechanism represents a critical bridge linking the immune microenvironment with the stromal-physical microenvironment and constitutes a key pathway through which circadian disruption, initially at the immune level, progressively reshapes the global landscape of the TME (Fig. 2).

### Stromal microenvironment

The stromal microenvironment includes CAFs, vascular endothelial cells, the extracellular matrix (ECM), and hypoxic conditions. These components are all regulated by circadian rhythms and collectively influence the malignant phenotype of CRC [41]. Consequently, tumor cells become highly sensitive to stromal pro-tumorigenic signals, particularly those mediated by the TENASCIN and THBS pathways, thereby enhancing invasive and metastatic potential.

Increasing experimental evidence indicates that vascular endothelial growth factor (VEGF) expression and secretion display circadian oscillations, which are driven by the cooperative activity of HIF-1α and BMAL1. Moreover, these rhythmic changes have been implicated in the regulation of angiogenesis [42]. In clinical CRC specimens, overexpression of hClock has been found to be positively correlated with advanced TNM stage and lymph node metastasis, and mechanistic studies suggest it promotes epithelial–mesenchymal transition (EMT) by activating the HIF-1α/VEGF axis [43]. At the ECM level, circadian rhythms regulate the synthesis and degradation of matrix proteins such as collagen, thereby influencing the dynamic stiffness of the ECM [44]. In Bmal1-knockout mouse models, loss of Bmal1 was shown to reduce plasminogen activator inhibitor 1 (PAI-1) expression, enhance fibrinolytic activity, and activate the TGF-β signaling pathway, leading to tumor fibrosis and the formation of the myCAF phenotype [45]. Hypoxia, a central physical feature of the CRC TME, has been shown to exhibit bidirectional interactions with circadian rhythms from cell-based assays. HIF-1α and BMAL1 display overlapping genome-wide regulatory targets, while hypoxic signaling can in turn slow the circadian cycle, forming a positive feedback loop [46]. Overall, each component of the stromal and physical microenvironment is under circadian regulation, and these components also influence one another. Factors secreted by CAFs promote angiogenesis, while ECM remodeling provides anchoring sites for CAFs and endothelial cells, and hypoxia simultaneously activates CAFs and promotes EMT.

**Figure 3:**
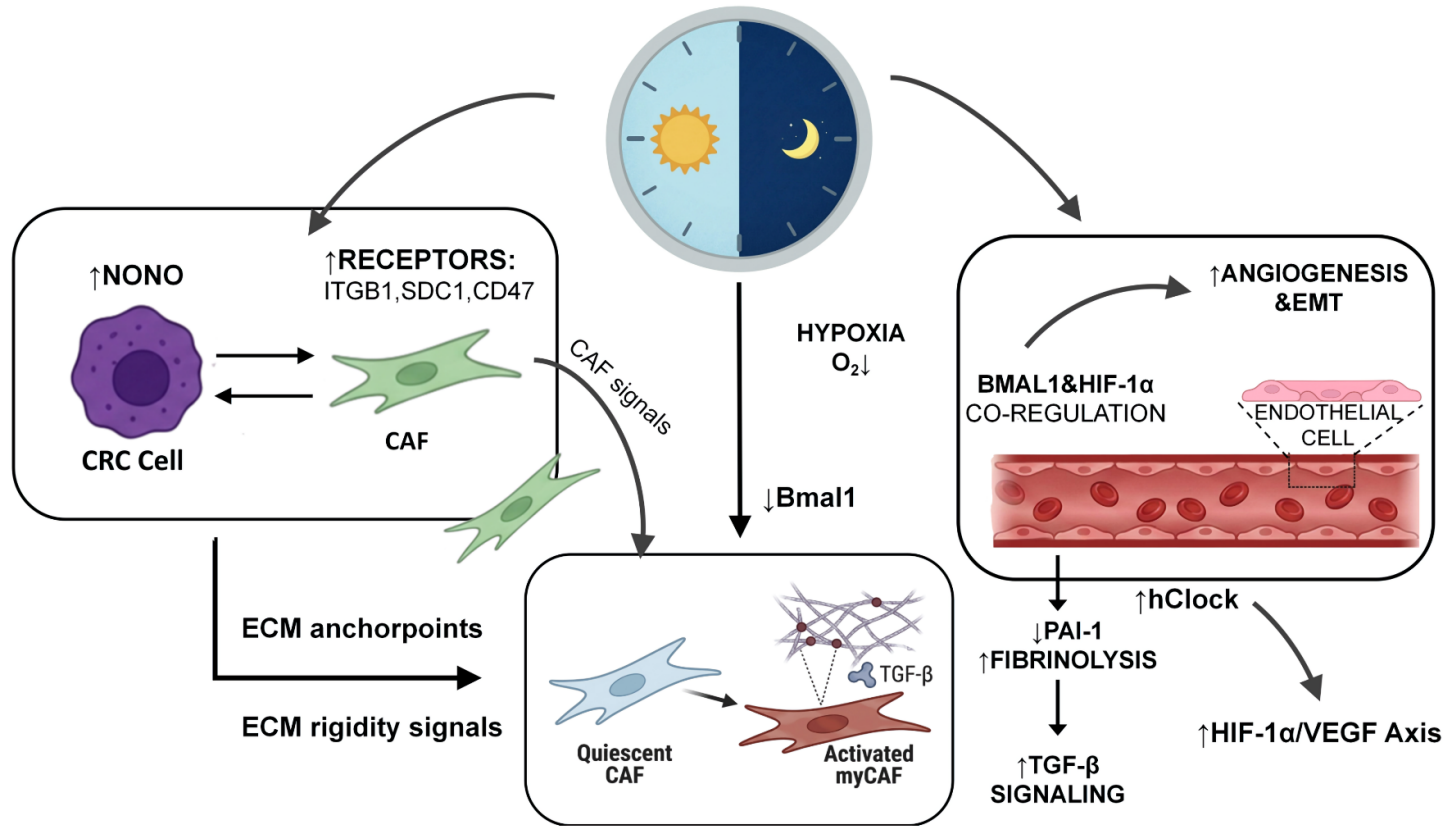
Circadian control of the stromal-physical microenvironment in CRC. Illustration of NONO-mediated CAF reprogramming and the HIF-1α/BMAL1-driven VEGF oscillation affecting ECM remodeling. BMAL1: brain and muscle ARNT like 1; CAF: cancer associated fibroblast; CRC: colorectal cancer; HIF-1α: hypoxia inducible factor 1 alpha; NONO: non-POU domain-containing octamer-binding protein; VEGF: vascular endothelial growth factor; EMT: epithelial–mesenchymal transition; ECM: extracellular matrix; TGF-β: transforming growth factor beta.

Metabolic abnormalities provide energy and biosynthetic substrates for tumor growth, immune dysfunction weakens antitumor immune responses, and structural abnormalities in the stromal and physical microenvironment create pathways and signaling support for tumor cell infiltration. These three aspects reinforce each other through positive feedback, forming a “metabolism-immunity-stroma” network. Circadian disruption acts as a key driving force within this coordinated system, pushing the TME from equilibrium toward imbalance (Table 1, Fig. 3) [24, 26–33, 35–37, 40–44, 46].

**Table 1:** Circadian Regulation of the Tumor Microenvironment in Colorectal Cancer

TME compartment	Key molecules/pathways	Cells involved	Role	Mechanism	References
Metabolic microenvironment	MYC/HIF-1α	Tumor cells	Promotes glycolysis, lactate production	Loss of clock genes (*BMAL1*/*PER2*/*CRY2*) activates this signaling axis.	[24, 27, 28]
	Lactate	CD8^+^ T, MDSC, TAM	Suppresses CD8^+^ T-cell activity; promotes MDSC/TAM immunosuppressive phenotypes	Lactate serves as a signaling molecule that directly modulates immune cell function.	[29, 30]
	Taurocholic acid (TCA)	MDSC	Enhances MDSC glycolysis; stabilizes PD-L1 expression	Circadian disruption → microbial metabolite accumulation → epigenetic glycolysis enhancement + PD-L1 ubiquitination inhibition	[31]
Immune microenvironment	PD-L1^+^ MDSC	MDSC	Forms immunosuppressive niche	Circadian disruption → altered epithelial clock → local cytokine changes → increased MDSC and neutrophil recruitment	[32]
	CD8^+^ T cells	CD8^+^ T cells	Antitumor immune exhaustion	Circadian disruption reduces infiltration and cytotoxicity; endothelial cells mediate oscillation	[33]
	Macrophages (M1/M2)	Macrophages	Phagocytosis, secretion, polarization fluctuate daily	Subject to phase-dependent circadian regulation.	[35, 36]
	SCFAs, BAs	CD8^+^ T, Treg, TAM, MDSC	Regulate immune cell function; affect vascular remodeling	Circadian disruption → altered gut microbiota metabolites → immune cell remodeling	[37]
	TGF-β, IL-6	CAFs, endothelial cells	Activate CAFs and promote myofibroblast phenotype; stimulate angiogenesis	Immune cells release TGF-β/IL-6 → CAF activation + angiogenesis, linking immune and stromal compartments	[26, 40]
Stromal microenvironment	NONO, ITGB1, SDC1, CD47	Tumor cells, CAFs	Enhance tumor cell receptivity to CAF-derived signals, promoting invasion and metastasis	NONO upregulates receptor expression, sensitizing tumor cells to TENASCIN/THBS pathways	[41]
	VEGF, HIF-1α, BMAL1, hClock	Endothelial cells, tumor cells	Promote angiogenesis, induce EMT, facilitate metastasis	HIF-1α and BMAL1 cooperatively drive rhythmic VEGF expression; hClock activates this axis	[42, 43]
	PAI-1, TGF-β, collagen	CAFs, tumor cells	Regulate ECM fibrosis and stiffness, promote invasion	Bmal1 loss → PAI-1↓ → fibrinolysis↑ → TGF-β activation → myCAF phenotype + fibrosis	[44]
	HIF-1α, BMAL1	Tumor cells, stromal cells	Form a hypoxia-positive feedback loop, sustaining pro-tumor microenvironment	HIF-1α and BMAL1 share overlapping genomic targets; hypoxic signaling in turn dampens circadian rhythmicity, thereby establishing a positive feedback loop	[46]

PD-L1: programmed death ligand 1; BMAL1: brain and muscle ARNT like 1; CAF: cancer associated fibroblast; HIF-1α: hypoxia inducible factor 1 alpha; integrin beta 1; NONO: non-POU domain-containing octamer-binding protein; PAI-1: plasminogen activator inhibitor 1; SCFA: short-chain fatty acid; SDC1: syndecan-1; TAM: tumor-associated macrophage; TGF-β: transforming growth factor beta; TME: tumor microenvironment; Treg: regulatory T cell; VEGF: vascular endothelial growth factor; EMT: epithelial–mesenchymal transition; IL: interleukin.

## Therapeutic Strategies

### Chronotherapy

Evidence indicates that aligning chemotherapy and immunotherapy with patients’ endogenous rhythms of drug-metabolizing enzyme activity and immune cell function can enhance antitumor efficacy while reducing toxic side effects, forming the evidence-based foundation for chronotherapy in CRC.

In the field of chrono-modulated immunotherapy, a meta-analysis of 11 clinical studies including 2,216 patients across multiple cancer types (including melanoma, non-small cell lung cancer, and renal cell carcinoma) demonstrated that individuals receiving immune checkpoint inhibitor (ICI) therapy between morning and midday (11:30 am–2:00 pm) had superior overall survival and progression-free survival (PFS) compared with those treated in the afternoon (3:00 pm–5:00 pm) [47]. However, the number of CRC patients included in this analysis was limited, and CRC-specific subgroup data were not separately reported, highlighting the need for dedicated chronotherapy studies in this population. Mechanistically, immune cell activity, migration, and infiltration have been shown to exhibit circadian oscillations in preclinical and clinical studies. Furthermore, a prevailing hypothesis posits that administering ICIs during the early phase of immune activity peaks could enhance synchronization with endogenous circadian rhythms and improve treatment outcomes. [32]. Incorporating patient chronotype as a low-cost individualized dosing parameter has been proposed as a potential strategy to further optimize ICI chronotherapy, although its specific clinical utility in CRC still requires validation through prospective randomized controlled trials [48].

The circadian hormone melatonin has also gained preliminary clinical and mechanistic support as an adjuvant therapeutic agent. Studies in murine CRC models have demonstrated that melatonin can reduce tumor volume by approximately 70%. Mechanistically, these studies have linked this effect to modulation of multiple signaling pathways, including NF-κB, PI3K/AKT, and Wnt/β-catenin, suppression of ROS, and regulation of clock genes such as *CLOCK* and *BMAL1* [23]. Additionally, a preclinical study has suggested that melatonin may reduce 5-fluorouracil resistance via the miR-532-3p/β-catenin axis [23]. The timing of melatonin administration is a critical determinant of its biological effects. Evidence from murine models demonstrates that melatonin administered in the morning can stimulate tumor growth, whereas administration in the late afternoon or evening exerts inhibitory effects [49]. In clinical practice, melatonin is most commonly administered in the evening before bedtime to align with its endogenous nocturnal peak and to mimic the physiological circadian rhythm of melatonin secretion. For instance, in perioperative settings for CRC surgery, 5 mg of melatonin has been administered the night before surgery followed by an additional 5 mg 2 h prior to surgery [50, 51]. In adjuvant therapy contexts, doses typically range from 10 to 40 mg/day administered in the evening. Moreover, epidemiological reports have suggested that melatonin supplementation may lower the risk of CRC by 23–41% among night-shift workers, while clinical studies have reported that it can reduce the incidence of radiotherapy-associated oral mucositis by 53%. However, large-scale phase III randomized controlled trials involving gastrointestinal malignancies are still lacking, optimal dosing regimens (10–40 mg/day) and administration timing remain unstandardized, and molecular heterogeneity (e.g., *KRAS* mutations) may limit treatment response in certain patient populations [23].

### Traditional Chinese medicine (TCM) interventions

TCM interventions in the treatment of CRC are mainly reflected in two aspects: time-based medication according to circadian principles and circadian regulation by natural products [52].

The TCM theories of “correspondence between humans and nature” and “midnight–midday ebb-flow” propose that the circulation of qi, blood, nutritive, and defensive energy in the human body fluctuates through different meridians at specific times of the day, and that the functional states of internal organs vary at particular time points. These concepts provide a theoretical basis for time-specific therapeutic interventions and exhibit intrinsic consistency with modern circadian biology [53]. Guided by the chronotherapeutic theory of TCM, a clinical study has reported that time-directed administration of spleen-strengthening and qi-tonifying prescriptions (a classic formula for invigorating the spleen and harmonizing the stomach) combined with chronomodulated chemotherapy, effectively alleviates chemotherapy-induced gastrointestinal symptoms after radical resection of CRC, with an observed post-treatment quality-of-life stability rate of 70.0% [54, 55]. Time-based therapeutic strategies guided by the midnight–midday ebb-flow principle have also demonstrated potential in reducing chemotherapy-related toxicities during postoperative rehabilitation in patients with gastrointestinal tumors [56].

At the level of basic research, increasing attention has been directed toward the regulatory effects of TCM-derived monomers on clock genes. Cell-based studies have shown that curcumin can influence the expression of clock-controlled genes by modulating the transcriptional activity of the BMAL1/CLOCK heterodimer and exhibits circadian phase-dependent effects, suggesting that its antitumor activity may be associated with modulation of the circadian system [57]. In particular, a review has systematically summarized multiple mechanisms through which plant-derived medicines inhibit the progression of CRC. It also noted that compounds such as curcumin, resveratrol, berberine, shikonin, and dihydroartemisinin may possess potential circadian regulatory effects in the treatment of CRC, although these findings are largely derived from foundational studies [58].

In clinical studies investigating the effects of TCM formulas and single compounds on CRC, several reports have described anticancer effects mediated through regulation of clock genes. For example, an experimental study has shown that the traditional formula Jiming San can activate the nuclear receptor RORα in colonic epithelial cells, thereby upregulating the expression of BMAL1 and PER2, restoring intestinal circadian rhythms, and suppressing NF-κB signaling pathway-mediated inflammatory responses [59]. In addition, TCM interventions from the perspective of gut microbiota have been increasingly investigated [60, 61] The multi-component characteristics of TCM formulas enable broader modulation of microbial communities, which may indirectly influence host circadian homeostasis [62].

### Behavioral interventions

Behavioral interventions consisting of stable light exposure patterns, scheduled meals, and regular physical activity represent fundamental approaches for restoring circadian homeostasis [63]. At the population level, a cohort study based on nearly 380,000 participants from the UK Biobank reported that the combination of healthy sleep patterns and healthy lifestyle behaviors was associated with a 28% reduction in overall cancer risk (hazard ratio (HR) = 0.72), including a 20% reduction in CRC risk (HR = 0.80) [64].

It is well established that a regular light-dark cycle maintains the normal rhythmic output of the SCN central pacemaker, thereby stabilizing the rhythmic expression of core clock genes such as *BMAL1* and *PER2* [65]. Experimental and emerging clinical evidence suggests that time-restricted feeding aligns the eating window with the active phase, indirectly influencing circadian oscillations of the gut microbiota and reducing the production of proinflammatory metabolites, thereby potentially improving the immunosuppressive state of the TME [66]. Collectively, this evidence indicates that behavioral interventions centered on regular sleep, light exposure, and eating schedules represent low-cost strategies for circadian regulation and may serve as beneficial adjuncts in lifestyle management and comprehensive treatment for CRC patients. However, their direct effects on tumor regression and the optimal timing for intervention require further investigation [63].

In addition to sleep duration and chronotype, comprehensive sleep hygiene practices [67]—including consistent bedtimes, avoidance of nocturnal light exposure, and regular meal timing—are essential for stabilizing circadian homeostasis (Table 2) [4, 23, 46, 66, 68–76].

**Table 2:** Clinical Evidence of Chronotherapeutic and Circadian-Based Interventions in Colorectal Cancer

Cancer type/stage	Intervention	Intervention group (summary)	Control group (summary)	Key significant differences (intervention vs control)	References
mCRC, stage IV	Chronomodulated chemotherapy	Chemotherapy infusion timed to circadian peaks (e.g., oxaliplatin, 5-FU)	Conventional constant-rate infusion (without chronomodulation)	Significantly reduced hematological toxicity (RR = 0.36; 95% CI, 0.27–0.48); objective response rate increased from 29% to 51% (P = 0.003)	[68]
	Chronomodulated FOLFOX	ChronoFLO4 regimen (4-day chronomodulated infusion)	FOLFOX2 regimen (2-day conventional infusion)	Significant benefit in males (HR = 0.75, P = 0.02), opposite effect in females (HR = 1.38, P = 0.03); overall survival comparable	[69]
	Chronomodulated chemotherapy + oxaliplatin	Chronomodulated 5-FU + leucovorin + oxaliplatin	Chronomodulated 5-FU + leucovorin (without oxaliplatin)	Objective response rate increased from 16% to 53% (P < 0.001); median PFS extended from 6.1 to 8.7 months (P = 0.048)	[70]
	Chronomodulated Chemotherapy + Irinotecan	Chronomodulated irinotecan infusion (peak at one of six different circadian time points) + fixed-schedule chronomodulated FOLFOX	Same regimen at different circadian time points	Significant sex differences (P < 0.05): morning administration recommended for males, afternoon for females	[46]
	Chronomodulated chemoradiotherapy	Chronomodulated concurrent chemoradiotherapy (radiation and chemotherapy timed according to circadian rhythms)	Conventional-timing chemoradiotherapy	Trends toward improvement in certain parameters; requires validation in larger studies	[23]
	Melatonin adjuvant therapy	Melatonin supplementation (standard dose, evening administration)	Placebo or no supplementation	23–41% reduction in CRC risk among shift workers; 53% reduction in radiotherapy-associated oral mucositis	[71]
Locally advanced rectal cancer (LARC, stages II–III)	Circadian rhythm and dietary intervention	Stable circadian rhythm + high dietary polyphenol intake	Standard care	Circadian rhythm stability and high dietary polyphenol intake associated with improved neoadjuvant therapy response	[72]
	Perioperative multicomponent behavioral intervention	Multicomponent behavioral program targeting circadian rhythm and gut–brain axis	Standard care	Associated with improvements in neuroimmune features and symptom outcomes	[73]
	Chronomodulated chemoradiotherapy	Chronomodulated chemoradiotherapy regimen (radiation timed according to circadian rhythm)	Conventional chemoradiotherapy	Faster recovery of peripheral blood white blood cell counts; fewer treatment delays; trend toward improved local control	[74]
Postoperative/adjuvant therapy and survivors (stages I–III)	Integrated sleep and psychological intervention	Sleep hygiene education + relaxation training + stimulus control + CBT	Standard oncological care	Significantly improved postoperative recovery and quality of life	[75]
	Behavioral activation	Behavioral activation intervention targeting sleep disorders	Standard care	Significantly improved sleep disturbances, quality of life, and psychological distress (P < 0.05)	[66]
	Circadian eating pattern intervention	Adjusted eating time windows and meal frequency	Conventional eating pattern	Longer energy intake window associated with less fatigue (P < 0.05)	[76]
High-risk populations (obesity/early-onset CRC)	TRE	8-h TRE (12:00 pm–8:00 pm)	Daily 25% calorie restriction or control group	Ongoing trial: assessing weight loss, metabolic improvement, and CRC risk reduction	[23]
	TRE + mindfulness intervention	8-week remote TRE (12:00 pm–8:00 pm) + mindfulness sessions	Standard care	Good feasibility and acceptability, providing basis for larger-scale trials	[4]

RR: risk ratio; CI: confidence interval; HR: hazard ratio; CBT: cognitive behavioral therapy; chronoFLO4: chronomodulated floxuridine-based 4-day regimen; CRC: colorectal cancer; FOLFOX: folinic acid + fluorouracil + oxaliplatin; LARC: locally advanced rectal cancer; mCRC: metastatic colorectal cancer; PFS: progression-free survival; TRE: time-restricted eating.

## Conclusions

This analysis consolidates current evidence that circadian disruption drives CRC progression through three interconnected dimensions of the TME. First, at the metabolic level, deletion of BMAL1, PER2 and CRY2 promotes MYC/HIF-1α-dependent glycolysis and lactate secretion, whereas gut microbial metabolites such as TCA accumulate under circadian control to stabilize PD-L1 on MDSCs. Second, immunological alterations upon circadian disturbance include expanded PD-L1^+^ MDSCs and CD8^+^ T-cell exhaustion, accompanied by rhythmic dysfunction of macrophages and cytokine axes. Third, in stromal tissues, circadian desynchronization drives an invasive-permissive niche via non-POU domain-containing octamer-binding protein (NONO)-dependent CAF remodeling, VEGF/HIF-1α/BMAL1-controlled angiogenesis, and PAI-1/TGF-β-mediated ECM reconstruction. Clinically, aberrant expression of PER3, NR1D1, and CRY2 has been associated with poor prognosis in CRC patient cohorts, supporting the translational relevance of these mechanistic findings.

Significant progress has been made in understanding how circadian rhythms regulate the TME in CRC; however, substantial challenges remain in translating mechanistic insights into clinical practice. The broader application of chronochemotherapy and chrono-immunotherapy is limited by the lack of reliable circadian biomarkers and standardized monitoring tools. In addition, heterogeneity in circadian phenotypes among patients complicates the design of large-scale clinical trials. Sex- and age-related differences in circadian regulation of CRC also remain underexplored, and future prospective studies with stratified analyses are needed to address these gaps. Future studies should integrate multi-omics analyses with artificial intelligence to construct personalized chronotherapeutic models, while also developing small-molecule modulators targeting clock components to directly correct immune–metabolic imbalances within the TME.

TCM interventions, such as time-based medication guided by the Ziwu Liuzhu theory and the use of natural products, have shown potential in modulating biological rhythms; however, the molecular mechanisms by which they exert antitumor effects through clock genes still require validation through high-quality basic and clinical research. Consistent with our finding [77], only by systematically elucidating the multidimensional interaction network among metabolism, immunity, and stromal components driven by circadian rhythms, and by establishing standardized systems for circadian rhythm assessment, can CRC therapy truly advance toward a precision chronotherapy paradigm based on individual biological rhythms.

## Data Availability

The authors declare that data supporting the findings of this study are available within the article.

## Abbreviations

5-FU: 5 fluorouracil
anti-PD-L1: anti programmed death ligand 1 antibody
BA: bile acid
BMAL1: brain and muscle ARNT like 1
CAF: cancer-associated fibroblast
CBT: cognitive behavioral therapy
chronoFLO4: chronomodulated floxuridine-based 4-day regimen
CI: confidence interval
CRC: colorectal cancer
ECM: extracellular matrix
FOLFOX: folinic acid + fluorouracil + oxaliplatin
HIF-1α: hypoxia inducible factor 1 alpha
HR: hazard ratio
IL: interleukin
ITGB1: integrin beta 1
LARC: locally advanced rectal cancer
mCRC: metastatic colorectal cancer
MDSC: myeloid-derived suppressor cell
NONO: non-POU domain-containing octamer-binding protein
OS: overall survival
PAI-1: plasminogen activator inhibitor 1
PD-L1: programmed death ligand 1
PER2: Period 2
PFS: progression-free survival
SCFA: short-chain fatty acid
SDC1: syndecan 1
TAM: tumor associated macrophage
TCA: taurocholic acid
TGF-β: transforming growth factor beta
TME: tumor microenvironment
TRE: time-restricted eating
Treg: regulatory T cell
VEGF: vascular endothelial growth factor
